# From narcissistic vulnerability to Hikikomori: depression and online–offline relational discrepancy among South Korean young adults

**DOI:** 10.3389/fpsyg.2026.1834967

**Published:** 2026-07-29

**Authors:** Chan Mi Lee, Soohyun La

**Affiliations:** Department of Counseling, Dankook University, Yongin, Republic of Korea

**Keywords:** Hikikomori, impact message inventory, interpersonal circumplex model, online–offline relational discrepancy, pathological narcissistic vulnerability

## Abstract

**Introduction:**

Social withdrawal (Hikikomori) has emerged as a significant public health concern among South Korean young adults. This study investigates how Hikikomori tendencies are associated with the interaction between internal psychological vulnerability and discrepancies between online and offline relational experiences.

**Methods:**

In a cross-sectional survey, data from 352 South Korean young adults (aged 19–34; 156 males, 44.3%) were analyzed using a moderated mediation model (PROCESS Macro Model 7). Measures included the Pathological Narcissism Inventory–Vulnerability scale (PNV), the 25-item Hikikomori Questionnaire, the 10-item Center for Epidemiologic Studies Depression Scale, and the Impact Message Inventory–Generalized Other, which assesses interpersonal experience along the dimensions of control and affiliation.

**Results:**

Depression partially mediated the association between pathological narcissistic vulnerability and Hikikomori, and this indirect pathway was significantly moderated by online–offline relational discrepancies in both the control and affiliation dimensions, with control discrepancy showing the stronger effect.

**Discussion:**

The findings suggest that Hikikomori among individuals with elevated narcissistic vulnerability may be associated not simply with digital engagement but with discrepancies in interpersonal experience across relational contexts. Distinguishing how control and affiliation discrepancies are differentially associated with risk may inform more targeted approaches for supporting socially withdrawn young adults.

## 1 Introduction

Hikikomori is generally defined as a state of severe and prolonged social withdrawal lasting at least 6 months, characterized by disengagement from most offline social activities, including school, employment, and interpersonal relationships (Teo and Gaw, 2010; Kato et al., 2020). This condition is distinguished from simple introversion or temporary social avoidance and is considered a serious form of social isolation that affects multiple functional domains of an individual’s life. Although the phenomenon was first described in Japan, an increasing number of studies have reported similar patterns of extreme social withdrawal in diverse sociocultural contexts, including Europe, North America, and the Middle East (Teo et al., 2015; Kato et al., 2019). The growing body of this research suggests that Hikikomori should no longer be understood as a culture-bound syndrome but rather as a “contemporary society-bound syndrome,” driven by the interaction between individual psychological vulnerabilities and global structural shifts (Orsolini et al., 2023; Kato et al., 2024).

South Korea provides a particularly relevant context for investigating these mechanisms. National reports estimate that approximately 540,000 young adults (approximately 5% of the young adult population) aged 19–34 experience substantial social isolation, with nearly half categorized as severely withdrawn (Cheong et al., 2022). This pattern reflects broader structural shifts, including prolonged educational trajectories and unstable labor markets, which, in the South Korean context, often manifest as intensified social competition and a rigid emphasis on outward success or “face” (Arnett, 2023; Kim, 2023; Young, 2023). In such a cultural milieu, where self-worth is frequently tethered to extrinsic achievements (Han, 2015), constant social comparison creates a high-risk environment for psychological distress and social disconnection. These pressures coincide with elevated levels of psychological distress among Korean youth, including high rates of depression and suicide compared with other OECD countries (Health Insurance Review and Assessment Service, 2023).

One psychological vulnerability that may contribute to Hikikomori is pathological narcissistic vulnerability (Hirashima, 2001; Pozza et al., 2019; Hayakawa and Kato, 2023). Contemporary models conceptualize narcissism as a dual construct consisting of grandiosity and vulnerability (Pincus and Lukowitsky, 2010). In contrast to narcissistic grandiosity, which involves overt self-enhancement and interpersonal dominance, narcissistic vulnerability is characterized by fragile self-esteem, hypersensitivity to evaluation, chronic shame, and a tendency to withdraw from relationships when self-worth is threatened (Pincus et al., 2014; Miller et al., 2018). From the perspective of self psychology, this vulnerability is fundamentally linked to intense selfobject needs, such that self-cohesion relies heavily on external validation and empathic mirroring (Kohut, 1977). When these needs are thwarted—as is common in hyper-competitive social contexts—individuals may experience a profound sense of self-fragmentation and internalize relational failures as evidence of their own inherent worthlessness (Ronningstam, 2011; Caligor et al., 2015).

Depression may represent a central psychological mechanism linking narcissistic vulnerability with social withdrawal. Empirical studies consistently demonstrate strong associations between narcissistic vulnerability, shame, and depressive affect (Miller et al., 2018; Poless et al., 2018). Unlike grandiosity, which is often linked to externalizing behaviors, vulnerability demonstrates a robust correlation with emotional constriction and internalized distress, which eventually develop into clinical depression (Miller et al., 2017; Jauk and Kaufman, 2018; Dashineau et al., 2019). Depressive symptoms are associated with reduced motivation for social engagement and increased avoidance of interpersonal interactions, both of which co-occur with progressive disengagement from offline relationships (Porter et al., 2019; Ike et al., 2020). Over time, this process may contribute to persistent patterns of social withdrawal characteristic of Hikikomori.

In contemporary digital environments, however, socially vulnerable individuals may seek alternative relational contexts online. South Korea’s advanced digital infrastructure provides a unique ecosystem in which individuals can maintain a basic existence and seek compensatory validation without physical contact (Han, 2015). Online interactions allow greater control over self-presentation and reduce the risk of immediate interpersonal evaluation, making them particularly appealing to individuals with fragile self-esteem (Casale and Banchi, 2020; Fegan and Bland, 2021). However, an over-reliance on digital channels for self-validation can further alienate individuals from their actual social reality, as the gratification derived from virtual rewards fails to integrate with their offline identity, often intensifying feelings of emptiness and tendencies toward reality avoidance (Sakurai et al., 2024).

The present study conceptualizes this phenomenon as online–offline relational discrepancy. This refers to inconsistencies in relational self-experiences across digitally mediated interactions and face-to-face interactions. For example, individuals may experience relatively higher perceived interpersonal control or acceptance in online environments while simultaneously experiencing powerlessness or low affiliation in offline relationships. This discrepancy represents more than a numerical difference in scores; it reflects a clinical state of intrapsychic conflict in which the individual’s self-experience is fragmented across contexts, hindering the maintenance of a cohesive identity. Such discrepancies may be associated with greater structural instability of the self inherent in narcissistic vulnerability, potentially co-occurring with an increased reliance on controllable online environments, a tendency to perceive offline interactions as threatening, and social withdrawal as a strategy for self-regulation.

To examine these relational dynamics, the current study adopts the Interpersonal Circumplex (IPC) framework, which conceptualizes interpersonal behavior along two fundamental dimensions: control (dominance–submission) and affiliation (warmth–hostility) (Kiesler, 1983; Leary, 1957/2004). Within this framework, narcissistic vulnerability has been associated with interpersonal patterns characterized by low perceived control and low affiliation, reflecting relational inhibition and coldness (Wright et al., 2009). These individuals often organize relationships asymmetrically, seeking intimacy to fulfill selfobject needs while remaining defensively detached from the independent personhood of others (Kohut, 1977; Pincus and Hopwood, 2012).

To capture interpersonal functioning across contexts, the present study utilizes the Impact Message Inventory–Generalized Other (IMI-GO) (Wagner et al., 1995; Woodward, 2025), a self-report adaptation of the IMI-C. Rather than assessing responses evoked by a specific individual, the IMI-GO captures the typical reactions individuals expect others to show toward them across everyday interpersonal situations, indexing a generalized self-with-others relational representation that reflects fundamental interpersonal characteristics (Wagner et al., 1995; Woodward, 2025). Because the IMI-GO is grounded in generalized relational expectations rather than tied to a specific interaction partner or medium, it is well suited to comparing interpersonal self-experiences across different relational contexts. Grounded in the interpersonal circumplex, the IMI-GO assesses two orthogonal dimensions—affiliation and control—and, by indexing anticipated rather than directly reported interpersonal traits, reduces the defensive distortions and response biases common in narcissistic populations (Kiesler, 1996; Pincus and Hopwood, 2012; Schmidt et al., 1999).

Building on these theoretical perspectives, the present study proposes a moderated mediation model explaining Hikikomori among South Korean young adults. By elucidating these mechanisms, this study aims to suggest that clinical interventions for Hikikomori should move beyond traditional social skills training and focus on integrating the patient’s disparate self-experiences across online and offline domains. Specifically, pathological narcissistic vulnerability is hypothesized to be associated with depressive symptoms, which are in turn hypothesized to be associated with social withdrawal. Furthermore, discrepancies in interpersonal experiences across online and offline contexts are hypothesized to strengthen this association.

The research hypotheses are as follows:

*H1*: Pathological narcissistic vulnerability will be positively associated with depression, and depression will mediate the relationship between narcissistic vulnerability and Hikikomori.

*H2*: Online–offline discrepancy in interpersonal impact messages will moderate the mediation pathway such that greater discrepancies will strengthen the association between narcissistic vulnerability, depression, and Hikikomori.

## 2 Methods

### 2.1 Participants and procedure

The sample consisted of 352 young adults aged 19 to 34 years ( M= 25.74, SD= 3.88), recruited nationwide in South Korea, of which 156 were male (44.3%) and 196 female (55.7%). Data were collected via an online survey platform beginning in September 2025, following the guidelines of the Institutional Review Board of Dankook University (Approval No. DKU_IRB-2025-05-060-002). After reviewing an information sheet describing the study’s purpose, procedures, and the voluntary, anonymous nature of participation, all participants provided written informed consent on screen and were informed of their right to withdraw at any time without penalty. Upon completion, they received a small gift voucher as compensation.

In addition to the standardized measures described below, participants completed behavioral screening items assessing the frequency and duration of reduced social contact. Participants reported whether they left home fewer than four times per week and, if so, for how long (less than 1 month; 1–3 months; 3–6 months; 6 months to 1 year; or longer). These items allowed the sample to be characterized against the behavioral criteria commonly used to define Hikikomori (Kato et al., 2020). Behavioral screening indicated that the sample spanned the full range of withdrawal severity rather than representing a uniformly withdrawn group: 48.0% of participants (*n* = 169) reported leaving home fewer than four times per week, and 17.3% (*n* = 61) met the behavioral Hikikomori criterion of reduced going-out persisting for at least 6 months. For reference, 54.5 and 55.7% of the sample exceeded the HQ-25 cutoffs of 42 and 40, respectively, and 43.8% met the CES-D10 threshold for depressive symptoms (Table 1).

**Table 1:** Behavioral and clinical screening characteristics.

Characteristic	*n*	%
Reduced going-out (< 4×/week)	169	48.0
Duration < 3 months	31	8.8
Duration 3–6 months	75	21.3
Duration ≥ 6 months^a^	61	17.3
HQ-25 ≥ 40	196	55.7
HQ-25 ≥ 42	192	54.5
CES-D10 ≥ 10	154	43.8

Percentages are based on the total sample (*N* = 352). Duration was unavailable for 2 participants who reported reduced going-out.

a Met behavioral Hikikomori criterion = reduced going-out (< 4×/week) persisting for at least 6 months; all 61 participants meeting this criterion also exceeded the HQ-25 screening cutoff (≥ 40). HQ-25 = 25-item Hikikomori Questionnaire; CES-D10 = 10-item Center for Epidemiologic Studies Depression Scale.

### 2.2 Instruments

Pathological Narcissistic Vulnerability was assessed using 20 items from the vulnerability subscales of the Pathological Narcissism Inventory (Pincus et al., 2009), validated in Korean by Yang and Kwon (2016). This measure evaluates fluctuating self-esteem, contingent self-worth, and self-hiding. Items were rated on a six-point Likert scale ranging from 0 (not at all like me) to 5 (very much like me). Higher total scores indicate a higher level of narcissistic vulnerability. In the current study, Cronbach’s α was 0.95.

To measure social withdrawal, the Korean version of the Hikikomori Questionnaire-25 (HQ-25) (Teo et al., 2018), validated by Je et al. (2022), was employed. It consists of 25 items across three subfactors: isolation, socialization, and emotional support. Responses were recorded on a five-point Likert scale (from 0 = not applicable to 4 = applicable), with seven items (4, 7, 10, 15, 21, 22, and 25) reverse-scored. Higher scores represent a stronger tendency toward social withdrawal. Cronbach’s α was 0.98.

Depressive symptoms were measured using the 10-item Center for Epidemiologic Studies Depression Scale (CES-D10) (Radloff, 1977), using the Korean version (Bae et al., 2020). Each item was scored on a four-point scale (0 to 3) based on frequency over the past week. Total scores range from 0 to 30; a score of 10 or higher indicates the presence of depressive symptoms. Internal consistency ( α ) was 0.91.

Discrepancies in the perceived interpersonal self across online and offline contexts were assessed using the Impact Message Inventory–Generalized Other (IMI-GO) (Wagner et al., 1995; Woodward, 2025), a 28-item self-report adaptation of the IMI-C (Kiesler et al., 1997), validated in Korea by Kim et al. (2012). Whereas the IMI-C measures the interpersonal impact of a specific target individual, the IMI-GO captures the typical reactions individuals expect others to show toward them in everyday social situations, indexing a generalized self-with-others relational representation (Wagner et al., 1995; Woodward, 2025). The inventory assesses two orthogonal dimensions of the Interpersonal Circumplex—Control (Dominance–Submissiveness) and Affiliation (Friendliness–Hostility)—across four subscales of seven items each. Items are rated on a four-point Likert scale (1 = not at all, 4 = very much). Participants completed the inventory twice. The two administrations used identical items (“When I am with them, people generally feel…”); the relational context was specified by a section instruction preceding each block, which directed participants to respond with respect to their online relationships in one block and their offline relationships in the other. This allowed direct comparison of generalized relational representations across the two contexts. Discrepancy scores were calculated as the difference between offline and online scores (Offline−Online) for each dimension. The reliability coefficients (*α*) ranged from 0.72 to 0.86.

### 2.3 Data analysis

Data were analyzed using IBM SPSS Statistics 24.0 and PROCESS Macro v4.2 (Hayes, 2022). First, descriptive statistics and Pearson correlation analyses were performed to examine the basic distributions and relationships among the study variables. Second, the mediating effect of depression was tested using PROCESS Macro Model 4. Third, the moderated mediation effects were tested using PROCESS Macro Model 7 (Hayes, 2022). For both the indirect and conditional indirect effects, statistical significance was determined via bootstrapping with 5,000 resamples to generate 95% bias-corrected confidence intervals (CIs); an effect was considered significant when the CI did not include zero (Preacher and Hayes, 2004; Preacher et al., 2007).

## 3 Results

### 3.1 Preliminary and descriptive analyses

As a preliminary step, and to provide a more intuitive depiction of between-group differences before examining continuous associations, participants meeting the behavioral Hikikomori criterion (*n* = 61; see Section 2.1) were compared with the remainder of the sample (*n* = 291). The withdrawn group reported significantly higher pathological narcissistic vulnerability (*M* = 63.62 vs. 44.07; *t* = 6.97, *p* < 0.001, *d* = 0.94) and depressive symptoms (*M* = 13.89 vs. 9.44; *t* = 3.78, *p* < 0.001, *d* = 0.66), along with a more pronounced online-favoring relational discrepancy, particularly in affiliation (AD: *M* = −3.77 vs. 0.33; *t* = −3.86, *p* < 0.001, *d* = −0.69; CD: *M* = −2.05 vs. −0.63; *t* = −2.04, *p* = 0.045, *d* = −0.31). These differences are consistent with the dimensional associations reported below; the main analyses treated Hikikomori as a continuous measure.

Table 2 presents the descriptive statistics and Pearson correlations among the study variables. The absolute values of skewness and kurtosis for all variables were below 2.0 and 7.0, respectively, indicating that the data met the assumption of univariate normality. Pathological narcissistic vulnerability was significantly and positively correlated with Hikikomori (*r* = 0.60, *p* < 0.01) and depression (*r* = 0.43, *p* < 0.01), and depression was strongly correlated with Hikikomori (*r* = 0.59, *p* < 0.01). Both discrepancy scores were negatively associated with Hikikomori (control discrepancy *r* = −0.16; affiliation discrepancy *r* = −0.37, both *p* < 0.01), and affiliation discrepancy was additionally associated with depression (*r* = −0.17, *p* < 0.01).

**Table 2:** Descriptive statistics and correlations among the study variables.

**Variable**	**PNV**	**De**	**H**	**CD**	**Ctrl_off**	**Ctrl_on**	**AD**	**Aff_off**	**Aff_on**
PNV	—								
De	.43**	—							
H	.60**	.59**	—						
CD	-.01	-.10	-.16**	—					
Ctrl_off	.06	-.20**	-.25**	.58**	—				
Ctrl_on	.07	-.08	-.04	-.62**	.27**	—			
AD	-.19**	-.17**	-.37**	.22**	.20**	-.07	—		
Aff_off	-.52**	-.32**	-.71**	.16**	.09	-.11*	.53**	—	
Aff_on	-.42**	-.20**	-.46**	-.02	-.08	-.05	-.31**	.65**	—
M	47.46	10.21	47.99	-0.88	-2.24	-1.37	-0.38	1.22	1.60
SD	22.14	6.88	26.73	4.63	3.76	3.91	6.12	7.62	6.82

*N* = 352. Values are zero-order Pearson correlations; leading zeros are omitted. Row and column labels correspond to the same variable. *PNV*, pathological narcissistic vulnerability; *De*, depression; *H*, Hikikomori; *CD*, control discrepancy; *AD*, affiliation discrepancy; *Ctrl_off*/*Ctrl_on*, offline/online control scores; *Aff_off*/*Aff_on*, offline/online affiliation scores. *p < .05. **p < .01.

To address the interpersonal indices separately, the offline and online component scores were included alongside the discrepancy scores in Table 2. For both dimensions, the offline indices were more strongly associated with Hikikomori than the corresponding discrepancy scores (offline affiliation *r* = −0.71; offline control *r* = −0.25, both *p* < 0.01), whereas the online indices showed weaker associations (online affiliation *r* = −0.46, *p* < 0.01; online control *r* = −0.04, *n.s.*). Offline and online indices were themselves moderately to strongly intercorrelated (control *r* = 0.27; affiliation *r* = 0.65, both *p* < 0.01).

Separately, because the discrepancy scores were computed as simple difference scores, which carry well-documented psychometric limitations (Edwards, 2002; Johns, 1981), supplementary polynomial regression and response surface analyses (RSA) were conducted as a robustness check, using mean-centered online and offline component scores (*N* = 352). For the control dimension, the quadratic terms added significant incremental variance beyond the linear terms (ΔR^2^ = 0.081, *p* < 0.001), indicating a nonlinear association between online–offline control discrepancy and Hikikomori that a simple difference score does not fully capture. For the affiliation dimension, the quadratic increment was not significant (ΔR^2^ = 0.008, *p* = 0.145). Full polynomial regression coefficients, response surface parameters, and plots are reported in the Supplementary material.

### 3.2 Mediation analyses

To examine whether depression mediates the relationship between pathological narcissistic vulnerability and Hikikomori, a mediation analysis was conducted using PROCESS Macro Model 4. Pathological narcissistic vulnerability had a significant positive effect on depression (*β* = 0.43, p < 0.001), and both pathological narcissistic vulnerability (*β* = 0.43, p < 0.001) and depression (*β* = 0.40, *p* < 0.001) significantly predicted Hikikomori. The total effect of narcissistic vulnerability on Hikikomori was also significant (*β* = 0.60, *p* < 0.001), and the inclusion of depression as a mediator increased the explained variance in Hikikomori from 36.1 to 49.4%. The indirect effect of narcissistic vulnerability on Hikikomori through depression was significant, with the 95% bias-corrected bootstrap confidence interval excluding zero (effect = 0.21, SE = 0.04, 95% CI [0.15, 0.29]). Because the direct effect of narcissistic vulnerability remained significant alongside this indirect effect, depression partially mediated the relationship between pathological narcissistic vulnerability and Hikikomori, supporting H1 (see Table 3).

**Table 3:** Summary of bootstrapped mediation analyses of the effects of PNV on H through depression.

Outcome	Mediation analysis paths	β	95% bias-corrected CI	
			Lower	Upper
**H**	Direct effect (PNV → H)	0.43**	-	-
	Indirect effect (PNV → De → H)	0.21**	0.15	0.29
	PNV → De	0.43**	-	-
	De → H	0.40**	-	-
	PNV → H	0.60**	-	-

PNV, pathological narcissistic vulnerability; De, depression; H, Hikikomori. CI, bias-corrected confidence intervals based on 5,000 bootstrapped samples. The 95% confidence interval for the indirect effect does not include zero. ∗∗p<.01 .

### 3.3 Moderated mediation analyses

To examine whether online–offline discrepancies in perceived self-concept moderated the indirect effect of pathological narcissistic vulnerability on social withdrawal via depression, moderated mediation analyses were conducted using bootstrapping. Two separate analyses were conducted for control discrepancy and affiliation discrepancy. All predictor variables were mean-centered prior to the analysis to minimize multicollinearity and improve the interpretability of the interaction terms.

**Figure 1:**
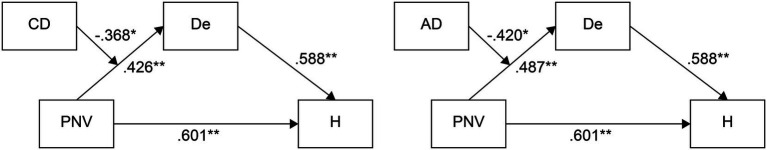
Proposed moderated mediation models: The moderating effect of CD (left) and AD (right). PNV, pathological narcissistic vulnerability; CD, control discrepancy; AD, affiliation discrepancy; De, depression; H, Hikikomori. Path coefficients in figure are conditional estimates from the moderated mediation models (Model 7) and may therefore differ from those of the simple mediation model (Model 4) reported in Table 3.

As per Table 4, first, control discrepancy significantly moderated the indirect association between pathological narcissistic vulnerability and social withdrawal through depression. The index of moderated mediation was significant (effect = 0.0 166,SE=0.0063 ), with the 95% bias-corrected bootstrap confidence interval not including zero (LLCI = 0.0048, ULCI = 0.0297). This finding indicates that the indirect effect of pathological narcissistic vulnerability on Hikikomori via depression became stronger as the discrepancies in perceived dominance–submission between online and offline relationships increased (see Figure 1, left).

**Table 4:** Moderated mediating effects of online–offline interpersonal impact message discrepancy in the association between PNV and H via depression.

Moderator	Interaction path	Effect	SE	95% CI LLCI	95% CI ULCI
CD	PNV × CD → De	0.0166	0.0063	0.0048	0.0297
AD	PNV × AD → De	0.0101	0.0057	0.0027	0.0247

N=352 . SE= standard error; PNV, pathological narcissistic vulnerability; CD, control discrepancy; AD, affiliation discrepancy; De, depression. CIs were estimated using bias-corrected bootstrapping with 5,000 resamples. A significant moderated mediation effect is indicated when the 95% confidence interval does not include zero.

Second, affiliation discrepancy showed a significant moderated mediation effect as well. The index of moderated mediation was significant (effect = 0.0101, SE = 0.0057) and the 95% bias-corrected bootstrap confidence interval did not include zero (LLCI = 0.0027, ULCI = 0.0247). This result suggests that the indirect pathway from pathological narcissistic vulnerability to social withdrawal through depression was likewise more pronounced at higher levels of discrepancy in perceived friendliness–hostility between online and offline relationships (see Figure 1, right). Taken together, these findings support H2.

Although both the control and affiliation discrepancies significantly moderated the indirect effect, the magnitude of the moderated mediation effect was relatively larger for the control discrepancy, suggesting that the depressive processes associated with pathological narcissistic vulnerability were more pronounced under conditions of greater inconsistency in perceived relational control.

**Figure 2:**
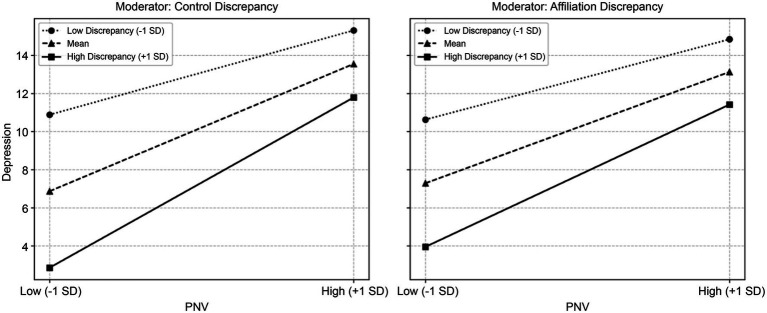
Depression as a function of PNV and control/affiliation discrepancy. PNV, pathological narcissistic vulnerability.

Figure 2 illustrates the nature of these interaction effects, showing that the association between pathological narcissistic vulnerability and depression becomes stronger at higher levels of online–offline interpersonal impact message discrepancy in both the control and affiliation domains.

## 4 Discussion

### 4.1 Interpretation of the main findings

The present study sought to clarify the psychological mechanisms underlying Hikikomori among South Korean young adults by examining the mediating role of depression and the moderating role of online–offline relational discrepancies measured through interpersonal impact messages. The findings are consistent with a moderated mediation model in which pathological narcissistic vulnerability is associated with Hikikomori via depressive symptoms, with this association stronger when online and offline relational experiences diverge.

These results supported H1: depression partially mediated the association between pathological narcissistic vulnerability and Hikikomori. The indirect effect of narcissistic vulnerability on Hikikomori through depression was statistically significant, and the direct effect of narcissistic vulnerability remained significant after accounting for depression, indicating that depression operates as a partial rather than a sole mechanism linking the two constructs. This finding is consistent with prior research identifying depression as a critical psychological pathway toward severe social withdrawal among emerging adults (Kim and Kim, 2018; Suwa and Suzuki, 2013; Loeffler et al., 2020; Pupi et al., 2025). Recent studies among Korean young adults similarly report a robust association between social withdrawal and depressive symptoms (Baek and Yoon, 2025; Park et al., 2025), and longitudinal evidence pointing to a bidirectional relationship between depressive symptoms and Hikikomori tendencies (Nonaka et al., 2026) further supports the clinical plausibility of the mediating pathway identified here. Together, these findings align with the broader literature suggesting that depression-driven withdrawal constitutes one distinct pathway within the heterogeneity of Hikikomori (Nonaka et al., 2025a, 2025b).

This mediating mechanism can be understood more specifically from a self-psychological perspective. Because the self-esteem of individuals with pathological narcissistic vulnerability is strongly contingent on external validation and evaluation (Pincus and Lukowitsky, 2010), dissatisfaction experienced in everyday interpersonal life or broader functioning is likely to be registered not as transient stress but as a threat to the self-structure itself. Importantly, such threats tend to be expressed not through externalizing behavior but through a withdrawn, passive, internalizing affect—namely, depression (Miller et al., 2018; Jauk and Kaufman, 2018; Dashineau et al., 2019). This is consistent with prior work contrasting narcissistic grandiosity, which is linked to externalizing behavior, with vulnerability, which is strongly associated with emotional withdrawal and internalized distress. The resulting depressive affect is characterized by diminished motivation and interpersonal retreat, features that are commonly observed alongside avoidance of social interaction and withdrawal from social roles—that is, Hikikomori (Porter et al., 2019; Ike et al., 2020). The present findings are consistent with this theoretical account.

Second, H2 was also supported. Online–offline relational discrepancy significantly moderated the pathway from pathological narcissistic vulnerability to Hikikomori via depression, and this moderating effect was confirmed for both the control and affiliation dimensions. This result is consistent with prior discussions of the so-called “paradox of connectivity,” whereby digital environments offer controllable, compensatory validation while simultaneously intensifying dissatisfaction with offline relationships (Kraut et al., 1998; Hu et al., 2022). That is, the present findings suggest that the critical issue is not increased online engagement per se, but rather the structural discrepancy between the online and offline relational contexts, with which the pathway from narcissistic vulnerability to depression, and subsequently to social withdrawal, was more strongly associated.

This moderating effect can be understood from a self-psychological perspective. To sustain a sense of self-cohesion, individuals with narcissistic vulnerability depend on selfobject experiences—that is, experiences of external validation and interpersonal influence (Kohut, 1977; Banai et al., 2005)—and online environments, which allow selective self-presentation and carry less immediate threat of negative evaluation, may relatively readily satisfy this need (Casale and Banchi, 2020; Fegan and Bland, 2021). When a comparable sense of control or recognition is not attained in offline relationships, however, a gap emerges between the controlled, compensatory online self-experience and the more fragile, unstable offline self-experience. This cross-context inconsistency in self-experience functions not merely as a difference between environments but as a psychological burden that is associated with diminished cohesion of the self-structure, and this pattern co-occurs with greater depression and withdrawal from offline relationships.

Comparing the moderated mediation effects of the two discrepancy variables, the moderating effect of control discrepancy was somewhat larger than that of affiliation discrepancy (see Table 4). This suggests that, of the two relational dimensions, cross-context discrepancy in control plays a relatively stronger role in the pathway from pathological narcissistic vulnerability to social withdrawal via depression, consistent with prior work linking narcissistic vulnerability to heightened sensitivity to control-related interpersonal signals (Pincus and Lukowitsky, 2010; Choi and La, 2025). The control dimension concerns agency—the exercise of influence and a sense of self-efficacy—and maintaining a consistent sense of influence across contexts constitutes an important basis for self-regulation (Kiesler, 1983, 1996; Wright et al., 2009). Given that the self-esteem of narcissistically vulnerable individuals is regulated in a manner that fluctuates with external conditions (Pincus and Lukowitsky, 2010), discrepancy in perceived control between online and offline contexts may be particularly relevant to disruption in this consistent sense of influence.

In this regard, the direction of the discrepancy carries an important theoretical meaning. The present study operationalized discrepancy as a signed difference (offline − online) because the risk signal for narcissistically vulnerable individuals was theorized to lie not simply in how far the two contexts diverge, but in an *online-favoring* discrepancy—a state in which needs for control and recognition that go unmet offline are relatively compensated within the more controllable online context. In such cases, a wider gap between the real self and the curated online self is associated with diminished self-cohesion, greater depressive affect, and greater withdrawal from offline relationships (Casale and Banchi, 2020; Fegan and Bland, 2021; Miller et al., 2018; Porter et al., 2019).

These results indicate that, when pathological narcissism is examined within the Interpersonal Circumplex framework, the agency and communion axes warrant separate rather than collapsed examination (Pincus and Hopwood, 2012). Although narcissistic vulnerability is often described as involving low control and low affiliation together (Wright et al., 2009), the finding that cross-context discrepancies in control and affiliation moderated the mediating pathway to differing degrees suggests that these two dimensions are better treated as linked to distinct relational processes than collapsed into a single composite index of interpersonal deficit.

This focus on cross-context discrepancy, rather than on the level of either context, rests on a deliberate theoretical stance regarding what the discrepancy is meant to capture. When examined separately, the offline component scores—particularly offline affiliation—showed stronger zero-order associations with Hikikomori than the corresponding discrepancy scores did (see Table 2). However, the theoretical interest of the present study lies not in the absolute level of control or affiliation within a single context, but in the cross-context discrepancy itself, which is theorized to be linked to instability of the self-structure of narcissistically vulnerable individuals. For the same reason, the relatively weak or nonsignificant zero-order correlations of the discrepancy variables with depression are not problematic, as a moderating effect operates through the interaction term rather than requiring a strong direct association between the moderator and the outcome.

### 4.2 Significance

This study carries significance on several fronts. Theoretically, by showing that cross-context discrepancies in control and affiliation moderate the pathway from narcissistic vulnerability to social withdrawal to differing degrees, it adds a more differentiated perspective to approaches that have treated these two interpersonal dimensions as a single composite index of deficit (Pincus and Hopwood, 2012). Rather than collapsing agency and communion into one index, the present findings suggest that the two axes may be linked to distinct relational processes and warrant separate examination. Methodologically, the study combines the context-separable IMI-GO with supplementary polynomial regression and response surface analyses, illustrating one analytic approach to examining cross-context discrepancy beyond simple difference scores while retaining the discrepancy as the construct of theoretical interest.

Clinically, the findings suggest that interventions for socially withdrawn young adults with narcissistic vulnerability should move beyond symptom-focused treatment of depression to address the relational mechanisms underlying withdrawal (Ronningstam, 2011; Caligor et al., 2015). In particular, therapeutic work may benefit from helping individuals explore both the deficits in agency they experience in offline relationships and their tendency to compensate for these deficits online (Casale and Banchi, 2020; Vogel et al., 2024).

Finally, because the mechanisms identified here were observed within the South Korean context, characterized by extensive digital infrastructure, the present study lends empirical support to recent perspectives that understand Hikikomori not as a culture-bound phenomenon but as one that may emerge across contemporary societies more broadly (Kato et al., 2024).

### 4.3 Limitations

This study has several limitations. First, the cross-sectional design precludes definitive causal inference, and the findings may have been influenced by biases inherent to survey-based research, including selection bias and limited generalizability. While pathological narcissistic vulnerability is conceptualized as a relatively stable personality-level vulnerability rather than a consequence of adult-onset withdrawal, the cross-sectional design cannot statistically rule out reverse or reciprocal pathways; longitudinal data are needed to clarify the temporal ordering among these variables. Second, although the IMI-GO provides a sophisticated measure of perceived interpersonal impact, it relies on individuals’ subjective perceptions, and all constructs were assessed via self-report within a single session. Although Harman’s single-factor test indicated no single dominant factor (first factor accounting for 26.9% of variance), this test is a relatively insensitive diagnostic (Podsakoff et al., 2003), and the possibility of common method bias cannot be fully ruled out. Future research should incorporate temporal separation, multi-informant designs, or observational approaches to capture interpersonal dynamics more objectively.

Third, beyond the conceptual rationale noted above for retaining the discrepancy score, the difference-score operationalization itself carries well-documented psychometric limitations, including reduced reliability and interpretive ambiguity (Edwards, 2002; Johns, 1981). The present study’s use of a signed rather than absolute difference reflected the theoretical position that the direction of the cross-context discrepancy is of substantive interest. To address this limitation, supplementary polynomial regression and response surface analyses, which showed differing patterns for the control and affiliation dimensions (see Section 3.1), should be understood not as a re-specification of the present model but as a robustness check on the psychometric substance of the difference-score representation: these analyses treat the online and offline scores as direct predictors of Hikikomori, which addresses a different question than the present model, in which discrepancy was specified as a moderator of the mediating pathway. The present study also did not measure the quantity of online engagement (e.g., daily time online). Future research could examine how usage intensity relates to online–offline relational discrepancy and to withdrawal. Finally, future studies should examine whether the mechanisms identified here vary across different types of online environments, such as anonymous communities, gaming contexts, or social networking platforms.

## 5 Conclusion

This study contributes to the growing literature on Hikikomori by clarifying a psychological pathway associated with severe social withdrawal among young adults with pathological narcissistic vulnerability. Depressive symptoms functioned as a partial mediating mechanism linking narcissistic vulnerability to Hikikomori tendencies, and this mediating pathway was significantly moderated by discrepancies between online and offline relational experiences in both the control and affiliation dimensions.

Importantly, the moderating effect of cross-context discrepancy was somewhat stronger for control than for affiliation, suggesting that inconsistency in perceived relational agency across online and offline contexts is particularly relevant within the pathway to Hikikomori among narcissistically vulnerable individuals. This pattern indicates that online–offline discrepancy should not be treated as a uniform risk indicator across relational dimensions, but as reflecting relational processes that may differ between the agency and communion domains.

Taken together, these findings suggest that Hikikomori may be understood not only as a phenomenon of social disconnection but also as a relational process reflected in how individuals’ interpersonal experiences of agency and recognition cohere—or fail to cohere—across online and offline contexts. Clarifying these distinct mechanisms may inform more targeted approaches to both clinical intervention and future research on severe social withdrawal among young adults.

## Data Availability

The raw data supporting the conclusions of this article will be made available by the authors, without undue reservation.
