# Supplementary material for: From narcissistic vulnerability to Hikikomori: depression and online–offline relational discrepancy among South Korean young adults
Source: Front Psychol. 2026 Jul 29;17:1834967. doi: 10.3389/fpsyg.2026.1834967 (PMC13462380; doi:10.3389/fpsyg.2026.1834967)
Supplement: Supplementary file 1 [file Table_1.DOCX]

# Supplementary Material

**Supplementary Text S1. Polynomial Regression and Response Surface Analysis of Online–Offline Interpersonal Discrepancy**

Because the online–offline discrepancy scores used in the main analyses were computed as simple difference scores (offline − online), which carry well-documented psychometric limitations (Edwards, 2002; Johns, 1981), we conducted supplementary polynomial regression and response surface analyses (RSA; Edwards, 2002) as a robustness check on the difference-score representation rather than as a re-specification of the main model. The online and offline component scores were mean-centered and entered as separate predictors of Hikikomori (*N* = 352). For each dimension, linear terms were entered at Step 1 and the quadratic terms (X^2^, XY, Y^2^) at Step 2. Four surface parameters were derived following Edwards (2002): the slope (a_₁_) and curvature (a_₂_) along the line of congruence (X = Y), and the slope (a_₃_) and curvature (a_₄_) along the line of incongruence (X = −Y); standard errors were obtained via the delta method. All analyses were conducted in IBM SPSS Statistics (Version 24.0).

For the control dimension (CD model), the quadratic terms added significant incremental variance beyond the linear terms (*ΔR*^2^ = .081, *p* < .001), and the curvature along the line of incongruence was significant (a_₄_ = 0.453, *p* < .001). For the affiliation dimension (AD model), the quadratic increment was not significant (*ΔR*^2^ = .008, *p* = .145). Full coefficients are presented in Table S1, the surface parameters in Table S2, and the fitted surfaces in Figure S1.

**Table S1.** Polynomial regression of Hikikomori on online and offline component scores (*N* = 352).

|  | **CD model (control)** | | | | **AD model (affiliation)** | | | |
| --- | --- | --- | --- | --- | --- | --- | --- | --- |
| Predictor | **Step 1** | | **Step 2** | | **Step 1** | | **Step 2** | |
|  | *B* | SE | *B* | SE | *B* | SE | *B* | SE |
| Online (X_c)_ | .173 | .369 | −.183 | .424 | −.024 | .194 | .017 | .206 |
| Offline (Y_c)_ | −1.794** | .383 | −.883* | .417 | −2.465** | .174 | −2.463** | .180 |
| Online^2^ | — | — | .200** | .063 | — | — | .055* | .026 |
| Online × Offline | — | — | −.037 | .075 | — | — | −.038 | .024 |
| Offline^2^ | — | — | .216** | .068 | — | — | .002 | .021 |
| *R*^2^ | .061 | | .142 | | .500 | | .507 | |
| *ΔR*^2^ |  | | .081** | |  | | .008 | |

*Note.* CD model = control discrepancy (online control × offline control); AD model = affiliation discrepancy (online affiliation × offline affiliation). All predictors were mean-centered. Step 1 entered linear terms; Step 2 added quadratic terms. — = not entered at that step. **p* < .05. ***p* < .01.

**Table S2.** Response surface parameters (*N* = 352).

|  | **CD model (control)** | | | | **AD model (affiliation)** | | | |
| --- | --- | --- | --- | --- | --- | --- | --- | --- |
| Parameter | Est. | SE | *t* | *p* | Est. | SE | *t* | *p* |
| a₁ slope, congruence | −1.065 | 0.477 | −2.233 | .026* | −2.446 | 0.169 | −14.505 | <.001** |
| a₂ curvature, congruence | 0.380 | 0.093 | 4.080 | <.001** | 0.018 | 0.016 | 1.138 | .256 |
| a₃ slope, incongruence | 0.700 | 0.692 | 1.011 | .312 | 2.480 | 0.348 | 7.120 | <.001** |
| a₄ curvature, incongruence | 0.453 | 0.116 | 3.917 | <.001** | 0.095 | 0.048 | 1.972 | .049* |

*Note.* Est. = estimate. a_₁_ = b₁ + b₂; a_₂_ = b₃ + b₄ + b₅; a_₃_ = b₁ − b₂; a_₄_ = b₃ − b₄ + b₅. The line of congruence is X = Y; the line of incongruence is X = −Y. Standard errors were computed using the delta method. **p* < .05. ***p* < .01.

**Figure S1.** Response surfaces for online–offline interpersonal discrepancy predicting Hikikomori tendency (*N* = 352). In each panel, the X axis is the mean-centered online component score and the Y axis the mean-centered offline component score; the vertical (Z) axis is the predicted Hikikomori tendency. The solid line marks the line of congruence (X = Y) and the dashed line the line of incongruence (X = −Y). (A) Control discrepancy (*ΔR*^2^ = .081, *p* < .001); (B) affiliation discrepancy (*ΔR*^2^ = .008, *p* = .145). Surface color denotes the predicted Hikikomori tendency, from blue (low) to red (high).
